# Initiation of traditional birth attendants and their traditional and spiritual practices during pregnancy and childbirth in Ghana

**DOI:** 10.1186/s12884-018-1691-7

**Published:** 2018-03-07

**Authors:** Lydia Aziato, Cephas N. Omenyo

**Affiliations:** 1 Department of Adult Health, School of Nursing, College of Health Sciences, University of Ghana, P.O. Box LG 43, Legon, Accra, Ghana; 2 College of Education, University of Ghana, Legon, Accra, Ghana

**Keywords:** Pregnancy, Traditional birth attendant, Traditional practices, Spirituality, Childbirth, Qualitative research

## Abstract

**Background:**

Prior to the advent of modern obstetric services, traditional birth attendants (TBAs) have rendered services to pregnant women and women in labour for a long time. Although it is anticipated that women in contemporary societies will give birth in hospitals and clinics, some women still patronize the services of TBAs. The study therefore sought to gain an in-depth understanding of the initiation of TBAs and their traditional and spiritual practices employed during pregnancy and childbirth in Ghana.

**Methods:**

The design was an exploratory qualitative one using in-depth individual interviews. Data saturation was reached with 16 participants who were all of Christian faith. Interviews were conducted with a semi-structured interview guide, audiotaped and transcribed verbatim. Content analysis was employed to generate findings.

**Results:**

The findings showed that TBAs were initiated through apprenticeship from family members who were TBAs and other non-family TBAs as well as through dreams and revelations. They practice using both spiritual and physical methods and their work was founded on spiritual directions, use of spiritual artefacts, herbs and physical examination. TBAs delay cutting of the cord and disposal of the placenta was associated with beliefs which indicated that when not properly disposed, it will have negative consequences on the child during adulthood.

**Conclusion:**

Although, TBAs like maternal health professionals operate to improve maternal health care, some of their spiritual practices and beliefs may pose threats to their clients. Nonetheless, with appropriate initiation and training, they can become useful.

## Background

Despite efforts to reduce maternal and infant mortality, low and middle income countries continue to report significant mortality rates, with some of the reasons being poor access to or low quality of professional care [1, 2]. In Africa, traditional birth attendants (TBAs) have historically been the major caregivers for women during childbirth [3, 4]. Like many low and middle-income countries, pregnant women in Ghana continue to either give birth at home or with TBAs [5]. A Traditional birth attendant (TBA), according to WHO is “a person who assists a mother during childbirth and who initially acquired her skills by delivering babies herself or through apprenticeship to other traditional birth attendants” [6].

In Ghana, traditional midwifery has been a part-time work for unskilled persons who mediate pregnancy and birth with some spiritual practices. Many TBAs rely on herbal medicines which are culturally inherited to assist women before, during and after labour [7]. Meanwhile, research suggests that these TBAs have had very little training and education that might integrate them into the larger health care system and even those with training need the support of skilled back up services [8, 9].

In 1987, introduction and adoption of safe motherhood programmes in Ghana drew attention to the need for women to patronize professional healthcare services during pregnancy and childbirth [10, 11]. However, these services are limited and not easily accessible or of low quality. In rural communities, over 30% of pregnant women do not have access to skilled birth attendants. Therefore, some of these women continue to access the services of TBAs. According to the Ghana Maternal Health Survey of 2014, 16% of deliveries were supervised by TBAs [12]. However, there is a dearth of information on the type of support and remedies they provide to women during pregnancy [13].

TBA care has been known to cut across pregnancy, labour, postpartum and care of the newborn [14]. Preference for TBAs has also been attributed to the fact that they provide affordable [15] and accessible services as well as conduct delivery at home- an environment familiar to the woman [16, 17]. Understanding and respect for the religious beliefs of clients is also associated with the preference for TBAs [18]. Furthermore, in a country like Ghana where health services are inadequate, the services of TBAs continue to be in demand [19, 20]. It is thus, observed that health policies that neglect the impact of TBAs would not be effective because some women still prefer home delivery and TBA services [5, 17]. Hence, an in-depth understanding of the initiation, traditional and spiritual practices of TBAs is relevant for policy making.

Previous authors report that initiation into TBA practice includes formal training by district health staff and organizations [21], sacred calling through dreams or visions [22] and inheriting or apprenticeship from close relatives such as mothers [3]. The apprenticeship has the duration of two to five years under a family member but one to two years when the trainer of the TBA is not a family member [23] probably because of commitment and paid training for those who are not family members. The findings also pre-suppose that TBAs learn on the job and hence may not benefit from scientific and standard processes of childbirth. The literature reports that TBAs keep the pregnancy status of a woman secret until signs of pregnancy are obvious in order to protect both mother and baby [24]. They also assess the vagina for cervical dilatation during labour and some listen to fetal heartbeats by positioning a bamboo on the abdomen [25]. Some TBAs place women in labour in a pounding mortar and when labour unduly delays, the woman is accused of concealing secrets such as infidelity and that labour will only progress after confession [25, 26]. Traditional birth attendants also use herbal medicine [27] to manage prolonged labour and retained placenta [28] but when overwhelmed by complications they refer to health facilities [29].

Some TBAs add spiritual practices to their care [19] with the belief that pregnant women are susceptible to spiritual attacks that can hinder successful outcome [30]. In view of this, before childbirth TBAs offer prayers [28] for effortless and safe birth [31]. Some believe that a jerk of a right arm or eye is an indication of uncomplicated labour but complications are anticipated if the left was involved [22]. For most cultures, placenta and other birth products are associated with rituals [32]. For instance, in some cultures people believe that the placenta is buried, burnt or thrown into a river just after childbirth because contact with vaginal blood could cause ill-health or premature death [33]. However, there is fear that when the placenta is disposed of inappropriately, evil people can use it to harm the baby [34].

Although, the literature presented some knowledge on the initiation and practices of TBAs from various contexts including Ghana, the authors of this study observed a need for further insight as most of the studies have inadequately explored the topic. It is noted that most authors emphasized the need for the training of TBAs to promote maternal and neonatal wellbeing [35, 36]. Improved care could reduce maternal and neonatal deaths [19]. The authors of this study anticipate that understanding the practices of TBAs will inform future training programmes that could enhance maternal care and wellbeing. Hence, this qualitative study was designed to explore the initiation of TBAs and the spiritual practices they employ during pregnancy and childbirth in Ghana.

## Methods

### Design

The study adopted an exploratory qualitative design to gain in-depth understanding of the initiation and practices of TBAs as well as spiritual influences of their initiation and practice. The qualitative design allows probing and further exploration of emerging findings and was deemed appropriate for the study [37, 38]. This design was useful because we did not use an existing theory or framework but rather we used probes to follow-up on participants’ responses. This process afforded a deeper understanding of emerging themes.

### Setting

The study was conducted in a rural community in the Greater Accra Region (Kasseh) with the participants drawn from an organized group in Kasseh which includes TBAs. The Greater Accra Region is the smallest region in Ghana and is made up of 16 administrative areas. It is bordered on the north by the Eastern Region, on the east by Lake Volta, on the south by the Gulf of Guinea, and on the west by the Central Region. According to institutional data, maternal mortality ratio has worsened in the Greater Accra Region since 1992 as compared to the other administrative regions in the country [39]. It is thus imperative to understand the basis for the unexpected outcome by examining quality of care provided to women in this region which includes TBAs services.

Kasseh is a major town located between Sege and Sogakope on the Accra-Aflao road. Kasseh has the biggest market in four districts (Ada West, Ada East, South Tongu and North Tongu) in its area. It is connected by road to the district capital, Ada-Foah and a town called Big Ada. Although it is the most easily accessible town in the district, poverty is widespread. Majority of the indigenous people are subsistence farmers using non-mechanized rain fed agriculture and the minority being fishermen and traders. They are also highly religious with the majority of the population being Christians.

This setting was chosen because its communities were mainly emerging developments with limited access to health facilities that provide pregnancy and delivery care. It was also deemed as the appropriate place to get the targeted participants as there is also an organized group of TBAs in the town. The group includes TBAs from rural communities within the district who were believed to have adequate experience in traditional practices during childbirth. It is called “Association of TBAs, Herbalists and Spiritual Healers”. The association was established with 83 members but currently has a membership of 42. That is, 16 males and 26 females. The group at the time of data collection was made up of Christian TBAs. The group was formed as a means of bringing together all TBAs, Herbalists and Spiritual Healers in the community to network and share ideas. Members of the association meet every third Monday of the month to discuss progress, shortfalls and other relevant issues pertaining to their practices.

### Sampling and data collection procedure

Using a purposive sampling technique, both males and females were recruited. To be included in the study, TBAs should have practiced for two years. Permission was obtained from the leaders of the associations to enable the researcher book appointments according to the meeting days of the groups. A trained research assistant who could speak the Ada language fluently assisted the first author as a translator during the interviews of participants who only spoke Ada language. The interviewer (first author) does not speak the Ada language fluently. Other interviews were conducted in Twi and Ga. Only one participant spoke English during the interview. The interviews lasted between 30 and 45 min. The interviews started with a general question such as: *‘Please tell me how you became a TBA’* and responses were probed. Follow-up questions such as: *‘Please tell me what you do for pregnant women when they come to you’*. In-depth understanding was achieved in this study and concurrent analysis helped in full exploration of emerging themes. Privacy was ensured during interviews and permission was obtained to record the interviews. The interviews were conducted in an enclosed place near the meeting grounds. Participation in the study was also voluntary.

### Data management and analysis

Interviews were transcribed in English and an expert in the local language who conducted the interviews checked the transcripts for accuracy. The research team read the transcripts several times to fully understand the perspectives of the participants. Concurrent analysis was undertaken using the techniques of content analysis. Inductive analysis processes were followed to develop themes and sub-themes since no theoretical framework informed the formulation of themes. The researchers independently coded the transcripts, grouped the codes and generated themes and sub-themes [40]. The themes and sub-themes were discussed among team members to ensure the data were faithfully captured. The data were subsequently managed using the NVivo software version 11. Relevant data were sifted to support themes and sub-themes and the findings were presented with supporting verbatim quotes from participants.

### Rigour

Rigour or trustworthiness of the study was achieved using a number of procedures. Emerging themes were further investigated in subsequent interviews (member checking) until saturation was achieved. The researchers undertook prolonged engagement with 16 participants and this ensured that the phenomenon under investigation was fully understood. Also field notes were taken to record non-verbal observations and decision trails during the study. Again, independent coding and checking of transcripts ensured that the data and analysis were credible. Identification codes were used to present verbatim quotes. The ID numbers were assigned chronologically as participants were recruited. For example TBA1M – TBA4M.

### Ethical considerations

Ethical clearance for the study was obtained from the Institutional Review Board of the Noguchi Memorial Institute for Medical Research at the University of Ghana. Informed consent was obtained from all participants and the data was anonymized. Participants consented to the use of data for teaching and publication. Participants were also made aware of their right to withdraw from the study at any given time.

## Results

### Demographic characteristics of participants

A total number of 16 TBAs participated in the study, four males and twelve females. Few males are engaged in midwifery/TBA; therefore, the number of males in this study depicts what exists in the general Ghanaian context [41]. They were aged between 31 and 80 years and had been TBAs between three and over 40 years. All participants were Christians as there were no members from another religion in the group. Out of the 16 participants, ten were married, five were widowed and one was divorced. The number of children they had ranged from 2 to 11. Ethnic backgrounds were Ada (11), Ashanti (2), Ga (1), Kwahu (1) and Bono (1). They conducted five deliveries per month on average. None of the study participants had any formal education similar to all members in the group at the time of data collection. Within the socio-cultural context, it is rare for educated individuals to be TBAs unlike what the literature has shown in Zambia where the district health office sometimes trains TBAs.

### Initiation of traditional birth attendants

Thirteen of the Traditional Birth Attendants (TBA) acquired skills of managing pregnancy and labour through training from a family member and apprenticeship from experienced TBAs whereas the others had spiritual revelations.

*My elder brother delivers women in the house so when he is going to deliver somebody, he calls me to come and observe.* (TBA10F); *…I was taught by my mum; so as she does it, I learn*. (TBA16F)

A participant claimed she received the revelation to be a TBA from her late mother after several encounters with her through dreams.

*…my mother worked as a TBA before she died. …One day I slept and dreamt that my mum and I were going to deliver someone. These kinds of dreams continued for about 12 months. So I asked a TBA and he told me that my mother wants the work she left for me to continue.* (TBA9M)

Another participant who was a pastor supporting a TBA with prayers stated:

*I used to pray for a TBA before she delivers women because I am a pastor…in some instances when she is delivering, she would ask me to observe. ...One day she travelled so there was nobody around to help the women deliver so I took it up and I’m still in it.* (TBA11M)

One TBA who was a hunter first, compensated for the killing of pregnant animals by assisting pregnant women during childbirth.

*I was a hunter... I was asleep one day when God revealed to me that I should not hunt for animals again but deliver women. This is because at times I killed some pregnant animals that I never knew were pregnant.* (TBA3M)

Two participants believed they received the calling whilst in their sleep and later they were confronted with the reality of assisting women to give birth and continued to do so since. One later underwent some basic training and became a certified TBA.

*I had two dreams that women came to me and I delivered them; on the third occasion, it happened physically; so I followed what I saw in the dreams and delivered them successfully. After that then we had some education so now I am a TBA.* (TBA5F)

*I did not learn it anywhere; it came to me in a vision…* (TBA4F)

### Spiritual and physical practices of TBAs

TBAs in this study continued to have spiritual revelations to direct them to manage women during pregnancy and labour. The participants used artefacts and different herbs during their work.

Some claimed they had spiritual directions to guide their management of women through *hearing voices*. They were clearly shown how to go about the delivery.

*When the person comes to me I look at the stomach and a spiritual voice directs me on what to do; …when I pray for the person, the Holy Spirit tells me the time the woman will give birth.* (TBA4F)

*…the spirit directs me as to what particular herb to use.* (TBA5F)

Some TBAs owned prayer camps (religious institutions that act as alternative hospitals for a variety of ailments in Ghana) where they prayed for expectant mothers and gave them physical and spiritual guidance concerning their pregnancies. Some did deliveries in their prayer centres while others did so in their houses or that of their clients.

*…since I stay in the camp, I get some revelations about the pregnant women where evil spirits prevent the baby from turning in the womb...* (TBA7F)

A TBA believed that prolonged labour was an indication that the baby was spiritually *locked* up in the womb.

*When the birth of the child delays, it could mean that the womb is locked by a spirit; after prayers everything is cleared and the child would come out quickly.* (TBA6F)

Some TBAs asserted that they had *“visions”* of pregnant women coming to give birth with them before they were physically brought.

*I see the women in a vision ready to give birth. …then when someone is brought in, she will look exactly like the one I saw in the vision.* (TBA15F)

Most TBAs perceived that obstructed and prolonged labour was a sign of infidelity or adultery which was considered a taboo or was a result of relationship problems with other people.

*... I told her “you have been unfaithful to your husband?” and she spoke the truth by letting me know what she did …after the confession, we prayed and I used the “anointing oil” on her before she had safe delivery.* (TBA3M)

Apart from the visions and revelations that some TBAs received, they also offered prayers and performed certain rituals for a safe delivery focusing on removing effects of evil spirits.

*I pray for her that God should help her deliver peacefully…..* (TBA12F)

*…it is not all the time that it is physical, sometimes too it is spiritual. My wife’s father had to do some rituals before she could deliver.* (TBA1M)

Thirteen out of the sixteen TBAs used a number of artefacts (substances or items that have been prayed over or blessed) in their practices with the belief that it lessened pain or enhanced delivery. Artefacts such as: blessed water, malt, milk, mixture of soda water and milk, anointing oil, a preparation of soap, a preparation of boiled water and oil, fresh ground okro, Ada salt and cassava leaves.

Some TBAs prayed over water and gave it to the pregnant women to drink.

*…you would be praying and if you are directed to pray over water for her to drink, you do that and the baby turns or positions well and the mother kneels and she delivers easily.* (TBA10F)

Also, the Ada salt was used to perform some rituals which were believed to enhance easy delivery.

*…my dad just brings Ada salt; I don’t know what he says but before he finishes his rituals, the woman just delivers.* (TBA1M)

Three TBAs used anointing oil:

*…we also anoint their forehead and the tummy with anointing oil and then pray before the process of delivery.* (TBA3M)

One TBA prayed over a bottle of malt for the women to drink after delivery:

*...when you give birth and I observe your condition is not stable; I would pray over a bottle of malt and ask you to drink it.* (TBA11M)

Another TBA prayed over a tin of milk and asked women to drink it during delivery especially when labour is prolonged.

*...in some instances, the baby delays in coming out; so, I buy one tin of milk, pray over it and when she takes it, she is able to give birth.* (TBA6F)

A TBA perceived labour pain to mean the presence of a *kernel-like* substance and until it burst, pain will persist. The TBAs gave the pregnant woman a mixture of soda water and milk to drink..

*When they are in pain, I make them buy a drink called “soda water” and I pour milk into it. The pain means there is something like kernel that must burst. When she drinks the soda mixed with milk, it bursts that thing and the pain stops.* (TBA4F)

A TBA prepared special soap that has been prayed over for the pregnant women for bathing to revert any challenges they faced.

*...sometimes when women come, I prepare special soap and pray over it for them to use to bath…..* (TBA5F)

Other TBAs gave a mixture of boiled water and oil in order to revive a distressed baby and give them strength.

*...In instances where the baby does not turn and dies, I make them buy sachet water which I boil and add oil to it for them to drink; when they drink the baby gains strength and get revived.* (TBA7F)

### Use of herbs/material objects

Some participants used herbs regularly in their practice. “*I boil herbs with the sap and bark of trees. I would give it to you to drink”* (TBA5F).

Some of the herbs and Turkey berries *(beduru)* were used by TBAs as *blood tonics* that they believed were effective.

*…we have our own blood tonic where we give specific leaves and beduru* (Turkey Berry) *some used to prepare Ghanaian dishes. After you give the person within sometime the person gets the blood.* (TBA1M)

There were times where under the directions of the spirit, TBAs prayed over cassava leaves and used it to heal sick pregnant women.

*…if a woman comes to me sick and I don’t have any medicine to help, after praying the spirit can tell me to plug cassava leaves and use it to bath the woman I give it to the person to bath with it or to drink.* (TBA1M)

Some TBAs said a slippery vaginal discharge was present during labour. Thus, they gave pregnant women fresh ground okro *(Abelmoschusesculentus)* which they have prayed over for the women to drink in order to excrete more of these discharges.

*...sometimes what we do is that, we grind fresh okro, pray over it and give to them to drink. If you are going to give birth something slimy comes out like okro from under the woman. After we have given you the okro, the baby turns…sometimes we shake the stomach and after 5 minutes the child will turn…….* (TBA1M)

Other TBAs used some herbs to prepare a solution for enema to enhance the delivery of the baby.

*...there is a herbal preparation for enema…if you know that the time is due but the baby is delaying…you give the woman enema and after about 5 minutes the baby will come out.* (TBA1M)

In addition, some TBAs attributed inactiveness of babies in the uterus to increased amniotic fluid. In the quest to remove the extra water, the TBAs gave pregnant women some herbal preparations believed to be diuretics.

*…if there is more water around the child, the child is not active. So I can give the woman a herbal drug which makes her urinate more and the child is able to turn.* (TBA13)

Some TBAs said their herbal preparations cannot be given in conjunction with the prescribed drugs from the hospital.

*…mine, when you drink it now you wait for about two or three hours before taking that of the hospital’s.*(TBA16F); *...when you go for the hospital drug and bring it, we will allow you to take all of it before you take my herbal medicine. I don’t allow them to mix so I can see the effect well.* (TBA13)

In cases of difficult or prolonged delivery, they ask the women to chew and swallow certain leaves with salt.

…*in some cases, the womb tightens and it becomes difficult for the child to come out. We will pluck a leaf, and wash it, then put a small salt on it then we give it to her to chew and swallow and immediately, the child comes out.* (TBA14F)

### Physical examination and practices during pregnancy and labour

The TBAs confirmed pregnancy by vaginal examination to feel for a lump. During difficult labour, the participants washed the abdomen with water and physically realigned the baby with breech presentation.

*When they come I put my hand inside to feel a lump there indicating that they are pregnant. After that the pregnancy grows; when they experience labour and they come for delivery, I wash the surface of their stomach with water. I sometimes try to straighten the baby up physically if not well positioned.* (TBA6F)

Most of the TBAs used their fingers to estimate the cervical dilatation during labour and predicted time of delivery based on their findings.

…*when I check and get 3 fingers then I have to give food meaning that the time is almost close* (TBA14F)

They also assessed the amniotic membranes or flesh of the baby if the membranes are ruptured with their fingers and asserted that the closer the membrane or flesh is to vaginal opening the earlier the woman would give birth.

*...when I check and the flesh around the womb is near the vagina or I see it’s near the vagina, it means she can give birth right away.* (TBA15F)

### Beliefs and practices regarding umbilical cord and placenta

A TBA reported that she checked and felt the umbilical cord for pulsations. If pulsations were felt, the cord would not be cut until the pulsations diminished. This is to allow the return of the ‘*spirit*’, believed to be in the cord, into the baby.

*…when the child is born we hold the cord and check if it is pulsating strongly; that means the child’s spirit has reduced and the spirit is in the cord and if the beating of the cord stops, the child will be screaming and crying then it means the cord is now dead and can be cut…* (TBA14F)

Few TBAs had clients with retained placenta and in such instances, they gave the woman a specific leaf to chew.

*....when the placenta delays, we have a leaf that we give to her to chew. She will chew that leaf and it helps the placenta to come out immediately.* (TBA14F)

Other TBAs placed a woman with retained placenta on plantain leaves to deliver the placenta.

*…we just cut plantain leaves and place it on the floor. When you place the woman on it, within 5 mins the placenta will come out.* (TBA1M)

After the delivery of the placenta, it was buried according to the custom of the individual.

*We don’t just bury placenta. We have directions for burying placenta because that is the destiny of the child.* (TBA1M)

Some TBAs were of the view that the way the burial of the placenta is done determined what the child becomes later in life.

*…when going to bury the placenta, the part that was cut from the child must point upwards. When we turn the cord part downwards, the child would become a prostitute in the future.* (TBA11M).

Some TBAs believed that they never encountered issues of retained placenta because they offered pregnant women malt and milk before the start of labour.

*Since I started this delivering, I have never delivered any child where the placenta delayed …this is because when the woman comes, before the delivery process, I give them malt and milk which prevents all these problems.* (TBA3M)

## Discussion

The finding that TBAs were initiated into their work through apprenticeship from family members or other experienced TBAs, spiritual revelation and dreams or visions corroborated other findings regarding initiation of TBAs or their acquisition of skills [22, 27, 28]. Given that most of the respondents had no formal education, it is imperative that TBAs are formally trained since such knowledge will enable them to recognise early signs of complications and refer early so that lives can be preserved [27]. Training programs should also be made simpler in order to facilitate easy understanding. Training of TBAs will also promote the use of standard procedures during pregnancy and labour and prevent infections and other related intrapartum and postnatal problems [42, 43].

Furthermore, the finding that spiritual directions or revelations guide practices of most TBAs resonated that of Adegoke et al. where the roles of TBAs and their spiritual practices during childbirth were linked [19]. The belief by some TBAs that voices originated from the “Holy Spirit” through prayers and directed them in their practice and in the use of herbs was attributed to the fact that the respondents were Christians. Such beliefs may not be held by non-christian participants. Revelations of evil acts that cause a baby’s inability to turn in the womb, are rooted in the predominant African belief that occurrences do not only have physical but also spiritual causes. The finding also confirmed the belief that pregnant women are susceptible to spiritual attacks targeting pregnancy destruction and poor delivery outcomes [27]. Majority of TBAs reported praying, fasting and performing certain rituals to counteract evil spirits or activities intending to cause negative results of pregnancy or delivery [19]. This finding was reasonable given that most respondents had received no formal training to enable them attend to obstetric emergencies. Also, since some pregnant women in Ghana believe in spiritual influences in pregnancy and childbirth [44], incorporation of spiritual activities could continue to attract pregnant women for their services. Hence, the need for training of TBAs to do proper assessment of women in labour is necessary so that the life of a woman and her baby is not jeopardized.

Findings on infidelity are consistent with the literature where the phenomenon is linked to prolonged labour, excessive pain during labour, caesarean section or even death [45]. Women suspected of infidelity were compelled to confess [27], using anointed oil for safe delivery. This was one of the reasons that women preferred symphysiotomy because they then still had a vaginal delivery [46]. When practices such as this persist, TBAs could miss the opportunity of timely referral of pregnant women to health facilities. Findings on the use of anointing oil and prayers during delivery are consistent with the literature [28, 39].

Use of artefacts by TBAs in our study are similar to that of Aziato et al., where Ghanaian women enumerated a number of artefacts used in pregnancy and labour [39]. Religion and societal norms have some influence on the TBAs’ belief systems as well as their practices [47] and could possibly explain the concurrent utilization of religious artefacts in the TBAs’ practices with respect to pregnancy and delivery.

The study confirmed that the use of herbs is embedded in the practices of TBAs [27]. While some boiled trees bark and sap, others prepared herbs to excrete water and trigger foetal activity and others also ground fresh okro to excrete discharges and realign malpresentation and enema to enhance delivery.

During prolonged labour some of the herbs were chewed with salt while others were used to improve the blood level of women [28]. These herbs when not well treated could serve as a source of infection. Nonetheless, some Ghanaians prefer herbal medicine because of the belief that it is effective and has no side effect [48]. Pregnant women may patronize TBAs to obtain herbs. However, health professionals do not use herbs routinely for fear that such herbs, for example *Cytisusscoparius,* may trigger preterm labour, rupture the uterus, and affect the unborn baby and mother [49, 50].

Most TBAs reportedly diagnosed pregnancy by feeling for an abdominal lump using their fingers as reported in other studies [25]. TBAs used their fingers to assess cervical dilatation and the amniotic membranes. Nevertheless, this is a concern because TBAs scarcely use examination gloves during these assessments or delivery raising a high risk of transmission of infections and the introduction of bacteria from the vagina to the fetus (chorioamnionitis). The TBAs could also handle more than one pregnant woman routinely and could therefore transmit infections from client to client [28]. The need for the provision of resources for TBAs emphasized.

Delayed cord clamping is considered an international best practice for improving maternal and neonatal outcomes [51]. This shows that although TBAs may not have scientific explanations for some of their practices and try to explain things in metaphysical terms, their practices are not entirely harmful as portrayed by some professional health practitioners. However, problems could result from their failure to recognize danger signs, their inability to implement simple evidence-based interventions for complications, and delayed referral [46]. It is also emphasized that the cord should be cut with sterile instruments; hence, TBAs should be educated on this to prevent infections. One challenge encountered by TBAs is birthing of the placenta and burial by the family or in their presence according to their customs. Hadwiger & Hadwiger [52] recorded similar beliefs where the spouse buried the placenta at the dripping spot of roof water so that the baby will grow to be intelligent and courteous. This suggests that TBAs understand and respect the religious beliefs of their clients and adhere to their requests. In relation to this, the assertion can be made that some women would still continue to seek the services of TBAs since they perceive them as people who share in their values and beliefs.

From the study, it is revealed that most TBAs engaged in much trial and error which includes many traditional interventions during delivery. This suggests that services provided by some TBAs in the Kasseh district do not have defined guidelines that determine when they cannot manage a complication and this may lead to late referral with fatal consequences [52, 53]. This again calls for the need of training TBAs to consider early referral in order to save lives. As compared to other studies where most participants were females [4, 8, 46], we recruited four males indicating that a general socio-cultural preference for female TBAs in sub-Saharan Africa although this is not speedily but gradually changing.

The study involved only TBAs of African Christian orientation and perhaps TBAs of other religious systems may have different experiences. Future studies should investigate TBAs with other religious backgrounds to corroborate findings of this study.

The limitation of this study relates to the involvement of only TBAs from the Christian faith because other TBAs from other religions may have different practices that were not captured. Therefore our findings may not apply to other religious groups and comparison should be done with caution. Bias was minimized in this study through the use of the same research instrument and verification of transcripts using an expert in Ada language. The authors concede that TBAs from other religions and those who have formal education could have different experiences. Thus, the findings from this study should be generalized with caution.

## Conclusion

The study revealed different approaches of initiation of TBAs and it was realized that whatever the initiation process, training was necessary to incorporate standard procedures in the care of women. The concurrent use of spirituality, herbs and the opportunity of women to observe or practice their beliefs such as the disposal of placenta attracted women to TBAs’ services. Within the socio-cultural context of Ghana where religiosity is a key component of the culture [44], TBA services will continue to flourish and the use of some spiritual approaches in tackling maternal health issues may never cease. It is therefore important to train TBAs and provide them with the necessary resources to deliver appropriate services during pregnancy and labour in a holistic way, with much emphasis on the areas they find challenging such as cutting of the umbilical cord. A stronger collaboration with health professionals is also necessary to enhance their work.

## Abbreviations

HIV: Human immuno deficiency virus
TBA: Traditional birth attendant
